# The relationship between pre-service kindergarten teachers’ professional identification and career adaptability: A chain mediation model

**DOI:** 10.3389/fpsyg.2022.1045947

**Published:** 2022-11-14

**Authors:** Tianqi Qiao, Zhanmei Song, Jie Huang, Jingfeng Yan, Xueying Zhang, Yixuan Wang, Cong Liu, Yang Wang

**Affiliations:** ^1^School of Education, Wenzhou University, Wenzhou, China; ^2^Institute of International Education, Wenzhou University, Wenzhou, China

**Keywords:** professional identity, career adaptation, teacher efficacy, self-leadership, pre-service kindergarten teachers

## Abstract

**Background:**

The healthy development of preschool education requires the support of stable and high-quality preschool teachers. However, there are still many deficiencies in preschool teachers in China at present. For pre-service kindergarten teachers, it is very significant to improve their career adaptability and enter professional positions smoothly. Numerous studies have found the effect of professional identification on the career adaptability of pre-service kindergarten teachers, but few studies have explored the potential influencing mechanisms among variables. On the basis of previous studies, this study explores the chain mediating effect of teacher efficacy and self-leadership on the relationship between professional identification and career adaptability of pre-service kindergarten teachers.

**Methods:**

Three hundred eighty-eight participants were recruited from two schools in Zhejiang Province. After screening, 377 questionnaires for pre-service kindergarten teachers were used for data analysis. The questionnaire included self-reported demographic information, professional identification, teacher efficacy, self-leadership, and career adaptability. We collected information on variables by using PISNS, TSE, RSLQ, and CFI, and analyzed the data using SPSS.

**Results:**

(1) Professional identification and career adaptability have a significant positive correlation. (2) The influence of occupational identification on the career adaptability of pre-service kindergarten teachers was carried out in three different ways: professional identification → teacher efficacy → career adaptability, professional identification → self-leadership → career adaptability, and professional identification → teacher efficacy → self-leadership → career adaptability.

**Conclusion:**

Teacher efficacy and self-leadership may mediate the relationship between professional identification and career adaptability of pre-service kindergarten teachers. This study highlighted the complexity of the link between preschool teachers’ professional identification and career adaptability. The paper also discussed the limitations and implications of this study.

## Introduction

Internationally, according to Sustainable Development Goal 4.2 (SDG 4.2), “Equal Access to Quality Preschool,” governments around the world are increasing funding for early childhood education resources to ensure that all children have access to quality early childhood education by 2030 (Rad et al., 2022). In China, with the successive introduction of China’s two-child and three-child policies, people’s demand for preschool education has become stronger and more prominent. *China’s Education Modernization 2035 plan* points out that it is necessary to popularize high-quality pre-school education, continuously improve the pre-school education management system, investment system, and kindergarten system, vigorously develop public kindergartens, and speed up the development of inclusive private kindergartens. The healthy development of preschool education requires the support of stable and high-quality preschool teachers. In March 2018, when answering a reporter’s question, Chinese Minister of Education pointed out that there was a huge gap among millions of preschool teachers, including childcare workers, in my country. The profession of a preschool teacher has been considered the next hot profession. However, the market gap and high turnover rate of preschool teachers are the current problems in China’s preschool education field (Hong et al., 2021). Preschool teachers are a crucial factor affecting the quality of early childhood education. As the reserve force on kindergarten teachers, pre-service kindergarten teachers (i.e., students majoring in preschool education) need to smoothly transition from student status to teacher status. This involves the focus of pre-service kindergarten teacher training (i.e., career adaptability).

Based on Career Construction Theory (CCT), career adaptability refers to an individual’s resources or self-preparation to cope with current and expected tasks in their career role and to adjust when the world of work changes (Niles, 2011; Savickas and Porfeli, 2012). Rottinghaus also define career adaptability as an individual’s ability to plan and adapt to changing career plans (Rottinghaus et al., 2005). Savickas proposes four components of career adaptability (Savickas, 2005): career concern, career control, career curiosity, and career confidence. Career concern is a person’s future direction, that is, the degree to which he/she values and participates in preparing for the future. Career control refers to autonomy, self-discipline, and responsibility in making career decisions. Career curiosity is to search for the direction of a match between the self and the world of work. And career confidence refers to the belief in one’s abilities to dealing with problems or overcoming obstacles and the expectation of success.

Career adaptability is critical for those who are facing a changing work environment and need to respond to new demands, especially college students who are about to start employment after graduation. Career adaptability can help college students quickly adapt to changes in psychological resources that affect their career development according to their developmental stages and environmental conditions. Career adaptability has a positive impact on the employment of college students. Individuals with high career adaptability will devote more of themselves to future jobs, perceive fewer professional obstacles, and be more capable of transforming their professional intentions into behaviors. Studies have confirmed that with higher career adaptability, college students have better employment status after graduation (Guan et al., 2014). In addition, paying attention to and improving the career adaptability of students majoring in preschool education in the training stage will not only help reduce the turnover rate of kindergarten teachers (Zhao et al., 2022, but also help improve their professional quality, which is crucial to the long-term development of the quality of early childhood education. Therefore, research on predictors of career adaptability for preschool teachers is critical for developing more comprehensive intervention and promotion approaches.

### Professional identification and career adaptability

Many recent studies have demonstrated that professional identification positively affects career adaptability (Negru-Subtirica et al., 2015; Guan et al., 2016; Haibo et al., 2018). Professional identification reflects the degree to which a person’s career adaptability to their self-concept (Johnson et al., 2006; Hekman et al., 2009). According to social identity theory (Tajfel and Turner, 2004), individuals with higher professional identity will have more positive evaluation of their career and higher work motivation. The high intrinsic motivation of occupation can encourage individuals to devote more energy to the development of relevant occupational abilities, actively explore occupation-related situations, better understand the future possibility of occupation, prepare for the occupation, and improve career adaptability (Guan et al., 2016). The stronger the individual’s sense of professional identification is, the more recognition they have of their future occupation in terms of cognition, emotion, attitude, and value, which will be more conducive to their adaptability to the new working environment in the future. Studies have suggested that a high level of professional identification will lead to higher career adaptability and more career success (Haibo et al., 2018). Therefore, professional identification may positively predict career adaptability.

### The potential mediating effect of teacher efficacy

According to Bandura’s self-efficacy theory, it is generally believed that teacher efficacy is the subjective judgment of teachers on teaching function and teaching ability (Bandura, 1977). Social cognitive career theory states that a person’s self-efficacy affects beliefs about job interests, goals, and behaviors. Individuals with high self-efficacy are more able to overcome challenging tasks. The higher the individual’s sense of teacher efficacy, the stronger the confidence in the work, and the more energy they can devote to the development of professional ability. Career adaptability is composed of the planning attitude, self-exploration, and environmental exploration. Therefore, individuals with higher teacher self-efficacy have stronger abilities in these three aspects and stronger career adaptability (Schunk and Pajares, 2002). In addition, teachers’ professional identification and teacher efficacy are important components of teachers’ inner cognition and emotion, and the two are closely related. Research has shown that teachers’ professional identification can positively predict teacher efficacy (Koumoundourou et al., 2012; Stringer and Kerpelman, 2014). According to the social identity theory (Tajfel and Turner, 2004)，the higher the level of individual professional identification, the more positive evaluation of their professional ability, the higher their self-efficacy, their confidence in future work, and their career adaptability will also increase (McLennan et al., 2017).

### The potential mediating effect of self-leadership

Self-leadership is a person’s ability to improve one’s performance through self-regulation, including cognitive, motivational, and behavioral strategies. The essence of these mechanisms is how people direct themselves to perform tasks that are naturally motivated and manage themselves to do work that must be done but are not naturally motivated (Manz, 1986). Studies have shown that self-leadership is a predictor of college students’ career adaptability (Park and Cho, 2020). Individuals with high self-leadership tend to stimulate themselves to adequately work with obstacles they face (Neck and Manz, 2010). College students with high self-leadership perceptions have higher career control and are willing to take responsibility for their own career development, so they have higher career adaptability (Şahin and Gülşen, 2022). In addition, a conceptual view of the self-leadership development process holds that self-leadership development requires attention to the individual’s sense of self-identity if it is to be most effective (Pearce, 2007). Identity is the source of meaning on which self-leadership operates. The higher the individual professional identity, the higher the level of self-leadership (Pearce, 2007).

### The potential chain mediating role of teacher efficacy and self-leadership

Reviewing the previous literature, most studies use teacher self-efficacy and self-leadership as two independent variables to explore the impact of each on professional performance (Kim and Sim, 2020). However, studies have found that teacher self-efficacy and self-leadership are positively correlated (Mansor et al., 2013; Lee and Kim, 2016). Individuals with higher self-efficacy are more confident in their abilities, and confident individuals are found to have higher self-control (i.e., the development of self-leadership skills). At the same time, it will in turn enhance their perception of efficacy (Ma et al., 2016). While people with low self-efficacy perceive tasks as more difficult, they are more likely to experience failure, depression, stress, and helplessness, and have lower levels of self-control and self-leadership (Van Dinther et al., 2011).

### The current study

**Figure 1:**
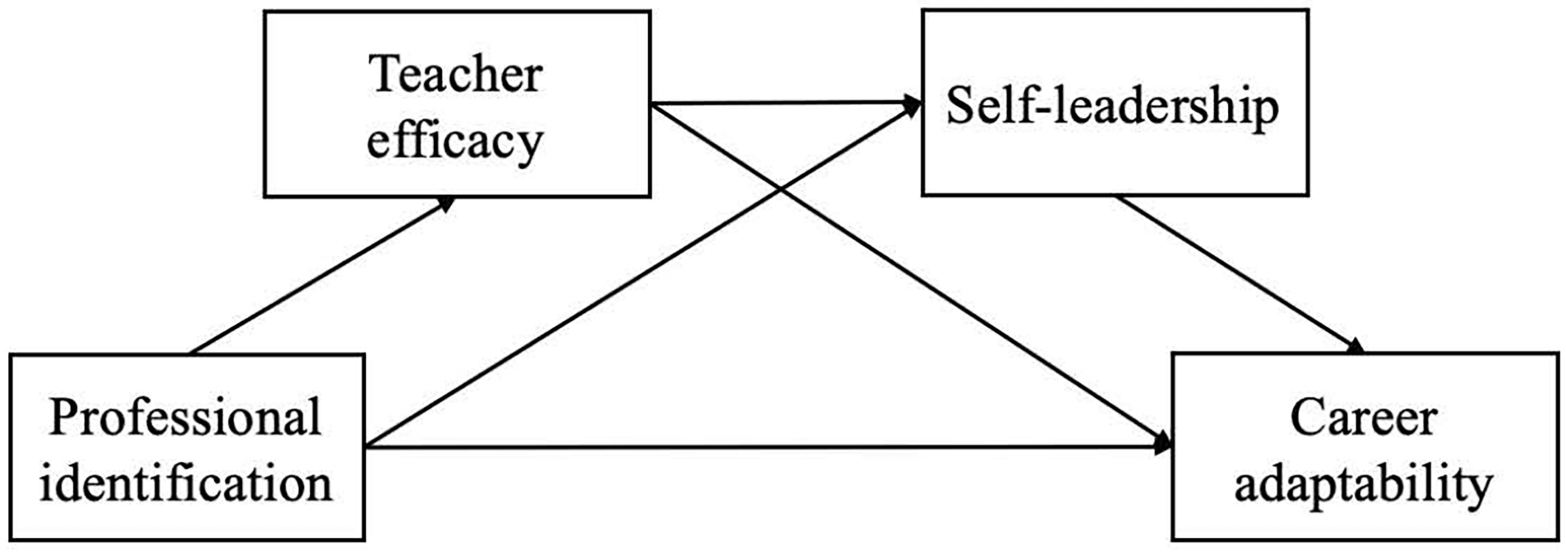
Hypothesized conceptual model.

At present, many studies have explored the correlation between teacher efficacy, self-leadership, professional identification and career adaptability, but few studies have explored their potential influencing mechanisms. This study took teacher efficacy and self-leadership as mediating variables, professional identification as independent variable, and career adaptability as dependent variable to explore their potential influencing mechanism. Considering the urgent need for the growth of pre-service kindergarten teachers, how to improve the career adaptability of pre-service kindergarten teachers becomes urgent. This study will explore the potential influencing mechanism of these variables, so as to provide reference for improving the career adaptability of pre-service kindergarten teachers. On the basis of previous studies, we propose the following hypothesis, and the theoretical hypothesis model is as follows (as shown in Figure 1):

*H1*: Professional identification can have a positive predictive effect on the career adaptability of pre-service kindergarten teachers.

*H2*: Professional identification indirectly predicts the career adaptability of pre-service kindergarten teachers *via* the mediating effect of teacher efficacy.

*H3*: Professional identification indirectly predicts the career adaptability of preschool teachers *via* the mediating role of self-leadership.

*H4*: Professional identification indirectly predicts the career adaptability of preschool teachers *via* the chain mediator of teacher efficacy and self-leadership.

## Materials and methods

### Participants and procedure

Data were collected from two universities of Zhejiang, China. Informed consent was acquired from all students and their teachers participated in the study. Ethical principles of confidentiality and voluntary participation were adhered. A total of 388 Chinese students majoring in preschool education filled in the questionnaire, and 377 valid questionnaires were returned, with a response rate of 97.16%. The data consisted of 365 female students (96.8%) and 12 male students (3.2%). Among them, 10 (2.7%) had technical secondary school degrees, 320 (84.9%) had college degrees, 45 (11.9%) had bachelor’s degrees, and 2 (0.5%) had master’s degrees. The mean age of the participants was 19.55 years (*SD* = 1.67).

### Measures

#### Personal information form

The personal information form includes participants’ age, gender and educational level.

#### Professional identification

We used the Professional Identification Scale for Normal Students (PISNS) to measure the professional identification of college students who major in preschool education (Wang et al., 2010). This scale has been used in previous studies (Zhu et al., 2019; Zheng et al., 2021). It was comprised of four subscales: professional expectation, professional volition, professional value, and professional efficacy. Example items include “I have the willingness to communicate with frontline teachers” and “I think teachers have a high social status.” Participants answer each item on a 5-point scale (1 = strongly disagree, 5 = strongly agree). Higher scores indicated higher levels of professional identity. This scale has adequate reliability and validity among Chinese (Zhu et al., 2019; Zheng et al., 2021). Cronbach’s alpha for this scale in the present study was 0.856.

#### Teacher efficacy

We adopted the Chinese version of the Short-form Teacher Efficacy Scale (TSE) to measure the teacher efficacy (Wu and Chim, 2017). The original scale (Tschannen-Moran and Hoy, 2001) consists of three dimensions. The Chinese version of the TSE is divided into two dimensions according to the localization results: efficacy for classroom management and efficacy for learning and teaching. Example items include “I can encourage students who are not interested in their studies” and “I can make students value learning.” The scale contains 12 items with scores ranging from 1 (not at all) to 9 (very much). Higher scores indicated higher levels of teacher efficacy. This scale has adequate reliability and validity among Chinese (Minghui et al., 2018). Cronbach’s alpha for this scale in the present study was 0.962.

#### Self-leadership

We used the Chinese version of the Revised Self-Leadership Questionnaire (RSLQ) to measure the self-leadership (Wang, 2014). The original questionnaire was developed by Houghton et al. (2002). The Chinese version of the RSLQ was comprised of nine subscales: success prediction, self-goal setting, self-talk, self-reward, belief hypothesis assessment, self-punishment, self-observation, natural reward and self-cue. Example items include “I use my imagination to picture myself performing well on important tasks” and “I visualize myself successfully performing a task before I do it.” The scale contains 35 items with scores ranging from 1 (strongly disagree) to 5 (strongly agree). Higher scores indicated higher levels of self-leadership. This scale has adequate reliability and validity among Chinese (Chen and Zhang, 2022). Cronbach’s alpha for this scale in the present study was 0.981.

#### Career adaptability

We used the Career Adaptability subscale of the Career Futures Inventory of the Career Futures Inventory (CFI) to measure career adaptability (Rottinghaus et al., 2005). Example items include “I am good at adapting to new work settings” and “I can adapt to change in the world of work.” The subscale contains 11 items with scores ranging from 1 (strongly disagree) to 5 (strongly agree). Higher scores indicated higher levels of career adaptability. This scale has adequate reliability and validity (Kim and Lee, 2018). Cronbach’s alpha for this scale in the present study was 0.841.

### Statistical analyses

SPSS 25.0 was used for descriptive statistics and correlation analysis of the data, and the model 6 of the SPSS macro program PROCESS provided by Hayes (Hayes, 2017) was used to test the mediating effect of teacher efficacy and self-leadership on professional identification and career adaptability. The bias-corrected bootstrap method was used to test the indirect effect. If the 95% confidence interval (CI) did not include 0, the indirect effect was significant.

## Results

### Common method biases tests

Since the self-reported scale was used for data collection in this study, common method bias may be caused. In order to control this effect, Harman’s single-factor test was used to statistically analyze the principal components of all items according to relevant research recommendations (Harman, 1976). The results showed that the variation explained by the first factor was 39.19%, which was less than the critical value of 40% (Podsakoff, 2003. Therefore, there is no serious common method bias in the data of this study.

### Descriptive and correlational analyses

The results of correlation analysis show that professional identification is positively correlated with teacher efficacy, self-leadership and career adaptability. Teacher efficacy is positively correlated with self-leadership and career adaptability. Self-leadership is positively correlated with career adaptability (as shown in Table 1).

**Table 1:** Descriptive analysis and correlations.

	Mean	*SD*	1	2	3	4
1. Professional identification	46.32	6.40	1.00			
2. Teacher efficacy	80.93	16.18	0.43^**^	1.00		
3. Self-leadership	136.36	21.88	0.59^**^	0.46^**^	1.00	
4. Career adaptability	40.59	5.87	0.60^**^	0.49^**^	070^**^	1.00

^**^p < 0.01.

### The chain mediating effects analyses

Professional identification, teacher efficacy, self-leadership and career adaptability are significantly correlated, which indicates that the mediating effect of teacher efficacy and self-leadership can be further examined (Wen and Ye, 2014).

**Figure 2:**
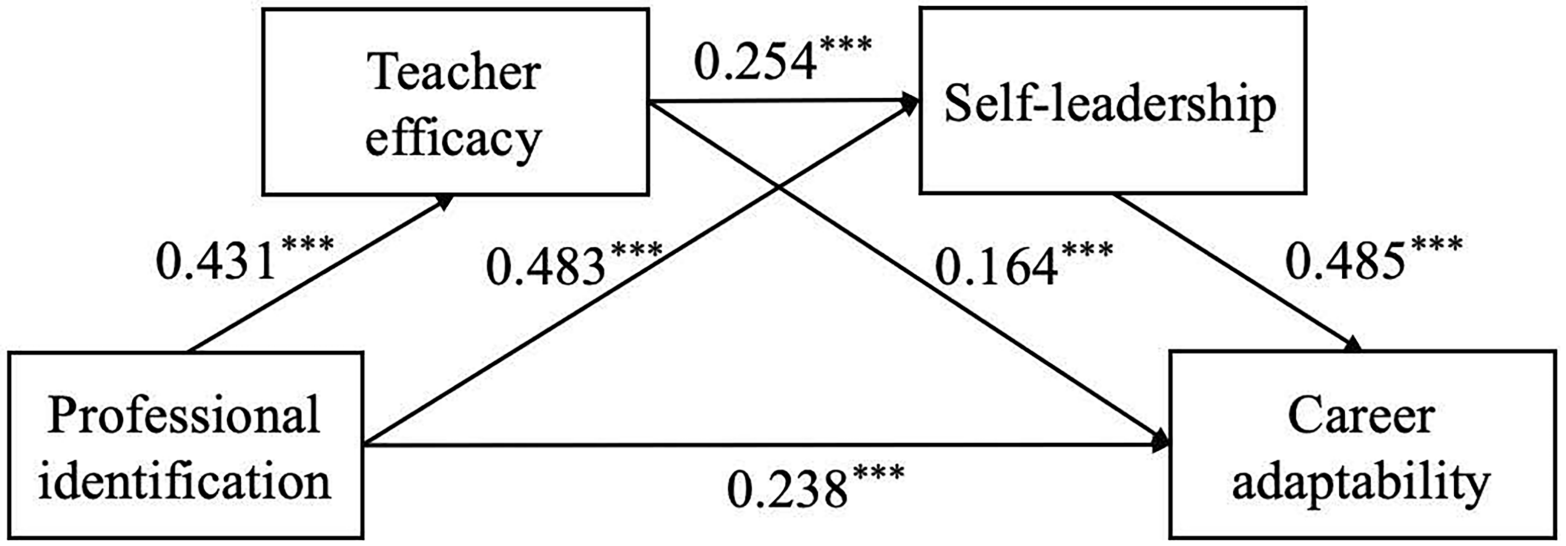
The chain mediating effect of teacher efficacy and self-leadership. ***p < 0.001.

All variables were standardized, and the mediating effects of teacher efficacy and self-leadership on the relationship between professional identity and career adaptation were tested according to the model 6 of the SPSS macro program PROCESS provided by Hayes. Gender, age and degree level were included as covariates in the regression equations. The regression analysis results are shown in Table 2, and the model diagram is shown in Figure 2. The total effect of professional identification on career adaptability was significant (β = 0.596, *p* < 0.001). Hypothesis 1 is confirmed. Professional identification significantly predict teacher efficacy (β = 0.431, *p* < 0.001) and self-leadership (β = 0.483, *p* < 0.001). Teacher efficacy significantly predict self-leadership (β = 0.254, *p* < 0.001) and career adaptability (β = 0.164, *p* < 0.001). Self-leadership is a significant positive predictor of career adaptability (β = 0.485, *p* < 0.001). These results indicate that teacher efficacy, self-leadership and the chain mediating effect of teacher efficacy → self-leadership are significant among the influences of life professional identification on career adaptability. Hypotheses 2–4 are confirmed.

**Table 2:** Regression analysis of the relationship between professional identification and career adaptability.

Regression equation		Fitting index			Significance	
Result variable	Predictor variable	*R*	*R* ^2^	*F*	*β*	*t*
Career adaptability	Gender	0.602	0.362	52.863	0.275	1.164
	Age				−0.047	−1.628
	Degree level				0.146	1.203
	Professional identification				0.596	14.377^***^
Teacher efficacy	Gender	0.433	0.188	21.500^***^	−0.179	−0.673
	Age				−0.035	−1.090
	Degree level				0.115	0.835
	Professional identification				0.431	9.202^***^
Self-leadership	Gender	0.640	0.409	51.407^***^	−0.202	−0.891
	Age				−0.027	−0.986
	Degree level				−0.043	−0.370
	Professional identification				0.483	10.897^***^
	Teacher efficacy				0.254	5.730^***^
Career adaptability	Gender	0.754	0.568	81.132^***^	0.424	2.176^*^
	Age				−0.023	−0.983
	Degree level				0.134	1.339
	Professional identification				0.238	5.477^***^
	Teacher efficacy				0.164	4.139^***^
	Self-leadership				0.485	10.912^***^

All variables in the model have been standardized. The control variables are gender, age and degree level, the independent variable is professional identification, and the outcome variable is career adaptability.

^*^p < 0.05; ^***^p < 0.001.

The bias corrected non-parametric percentile Bootstrap method was used to test the mediating effect by repeated sampling 5,000 times, and the results are shown in Table 3. The 95% confidence intervals of the three mediation paths did not include 0, and the three mediation effects were all significant. The total indirect effect value of professional identification on career adaptability is 0.385. The mediating effect accounted for 59.97% of the total effect. The mediating effect consists of three paths: path 1: professional identification → teacher efficacy → career adaptability (0.071), path 2: professional identification → self-leadership → career adaptability (0.234), and path 3: professional identification → teacher efficacy → self-leadership → career adaptability (0.053). Hypotheses 2–4 are reconfirmed.

**Table 3:** Teacher efficacy and self-leadership in the mediation effect analysis.

	Indirect effects	Boot SE	Boot LLCI	Boot ULCI	Relative mediation effect
Total indirect effect	0.358	0.050	0.264	0.458	59.97%
Indirect effect 1	0.071	0.027	0.028	0.130	11.83%
Indirect effect 2	0.234	0.042	0.150	0.317	39.26%
Indirect effect 3	0.053	0.018	0.023	0.092	8.89%

LL, lower limit; CI, confidence interval; UL, upper limit.

Indirect effect 1: professional identification → teacher efficacy → career adaptability; indirect effect 2: professional identification → self-leadership → career adaptability; indirect effect 3: professional identification → teacher efficacy → self-leadership → career adaptability.

## Discussion

By using a chain mediation model, this study aimed to analyze the association between professional identification and career adaptability of pre-service kindergarten teachers. The results of this study suggested that teacher efficacy and self-leadership played a partial mediating role in the correlation between pre-service kindergarten teachers’ professional identification and career adaptability. And our study further confirmed the direct link between preschool teachers’ professional identification and career adaptability (Negru-Subtirica et al., 2015; Guan et al., 2016; Haibo et al., 2018). The results showed that professional identification affects career adaptability through three paths: teacher efficacy, self-leadership, and teacher efficacy → self-leadership. This will help us to further comprehend the association between professional identification and career adaptability, and it will also help schools adjust training plans appropriately to further enhance the career adaptability of pre-service kindergarten teachers.

In this study, we first verified the mediating role of teacher efficacy on professional identification and career adaptability, which was consistent to a certain extent with previous research, that is, the partial mediating role of teacher efficacy in the relationship between professional identification and career adaptability (McLennan et al., 2017). In detail, we found that pre-service kindergarten teachers who had higher professional identification with themselves would be more confident in their professional ability, their teacher efficacy will be higher, and their career adaptability to future work will also increase. However, if the pre-service kindergarten teachers have a low degree of recognition of their majors during school, their understanding and learning effects of professional courses and training goals will be reduced, and their self-confidence in their professional abilities will also decline, ultimately leading to lower career adaptability.

Besides, this study identified important pathways for self-leadership, professional identity, and career adaptability. This model suggested that self-leadership played a partial mediating role between professional identification and career adaptability. A large number of studies have shown that the higher the individual’s professional identification, the higher the level of self-leadership (Pearce, 2007), and college students with high self-leadership have higher career adaptability (Şahin and Gülşen, 2022). That is, pre-service kindergarten teachers who had a higher identification with their own career were more likely to motivate and lead themselves to complete career-related tasks, actively deal with obstacles encountered in the process, and were willing to take responsibility for their own career development, therefore their career adaptability became higher.

This study also uncovered important pathways for professional identification, teacher efficacy, self-leadership, and career adaptability. The model suggested that the chain relationship between teacher efficacy and self-leadership mediated the relationship between professional identification and career adaptability. Many studies have shown that the higher the level of an individual’s professional identification, the higher the efficacy (McLennan et al., 2017), and teacher efficacy and self-leadership were positively correlated (Mansor et al., 2013; Lee and Kim, 2016). The higher the professional identity level of pre-service kindergarten teachers, the higher their teacher efficacy. In addition, individuals with higher self-efficacy would have higher self-control and self-leadership, thus having better career adaptability. This model showed that improvements in self-leadership were associated with teacher efficacy. When pre-service kindergarten teachers had higher self-efficacy, they were more confident in their careers, had more control over career-related tasks, and had higher self-control, which increased the level of self-leadership and in turn improved individual career adaptability.

Governments around the world are starting to work in a hierarchical early childhood education model at the micro (child, family and community), meso (nurseries and kindergartens) and macro (national policies and development goals) levels to ensure that all children have access to quality early childhood development and preschool education by 2030, to achieve Sustainable Development Goal 4.2 (SDG 4.2) (Rad et al., 2022). Preschool education is the foundation of lifelong education. And a stable and high-quality teacher team is an important guarantee for the sustainable development of preschool education. However, there is a huge gap in the current teaching staff, and there is an urgent need for good teachers to join in. As the reserve army of the teaching staff, it is very important for pre-service kindergarten teachers to enter the teaching staff smoothly. At present, kindergarten teachers face problems such as low wages, high work pressure, and a lack of corresponding institutional guarantees. Although, in recent years, the state has attached great importance to the high-quality development of preschool education, and has successively issued important policies to support it. However, due to the weak foundation of preschool education in China, the improvement of the social status and salary of kindergarten teachers is slow. Facing uncertain career challenges in the future, pre-service kindergarten teachers urgently need to improve their professional abilities and take the initiative to adapt to the environment. Because only with good career adaptability, can work stability and professional development be achieved. Besides, our study found that the higher the professional identification of pre-service kindergarten teachers, the higher the teacher efficacy, the higher the self-leadership, and the higher the level of career adaptability when completing the work. Therefore, for the training of pre-service kindergarten teachers, schools can consider joining courses to improve teacher efficacy and self-leadership or holding regular lectures to improve teachers’ efficacy and self-leadership. In addition, mindfulness training can also improve teachers’ efficacy and self-leadership, which in turn can improve their level of career adaptability (Furtner et al., 2018; Sharma and Kumra, 2022).

## Limitations and conclusion

However, this study has certain limitations. First, as a cross-sectional study, the results only show correlations between variables, not causality. In the follow-up study, the longitudinal study method can be considered. Second, all the questionnaires collected in this study were self-reported questionnaires, and participants may be influenced by social expectations. The follow-up research can adopt various methods such as classmate evaluation and teacher evaluation. Third, the samples of this study are only selected from two universities in Zhejiang Province, which has sampling constraints. Subsequent research can select samples from the whole country and even countries. Finally, this study only discussed the potential influence mechanism among the four main variables, but does not analyze in detail the effects of some possible moderator variables, such as family socioeconomic status, parental occupation, and so on, on the career adaptability of pre-service kindergarten teachers. Subsequent research can further explore and analyze some demographic variables that may affect the two mediating factors of teacher efficacy and self-leadership.

Despite the above limitations, this study is the first to examine the relationship between professional identification, teacher efficacy, self-leadership, and career adaptability with pre-service kindergarten teachers. We explored the mediating role of teacher efficacy and self-leadership in the relationship between professional identification and career adaptability and their cascading mediating effects. The findings supported our four hypotheses, namely, that professional identification had a positive predictive effect on the career adaptability of pre-service kindergarten teachers. At the same time, professional identification indirectly predicted the career adaptability level of pre-service kindergarten teachers through the mediating role of teacher efficacy, self-leadership, and the chain mediating role between teacher efficacy and self-leadership.

## Data availability statement

The original contributions presented in the study are included in the article/Supplementary material, further inquiries can be directed to the corresponding author.

## Ethics statement

The studies involving human participants were reviewed and approved by Wenzhou University. The patients/participants provided their written informed consent to participate in this study.

## Author contributions

ZS: conceptualization. TQ: methodology, validation, formal analysis, and writing—original draft. JH: writing—review and editing and project administration. JY: conceptualization and methodology. XZ: writing—original draft. YW: data curation. YW: writing-review and editing. CL: conceptualization. All authors contributed to the article and approved the submitted version.

## Funding

This study was funded by the Research on Development Assessment and Education Promotion of Left-behind Children based on Multimodal Information Technology (BHA160085).

## Conflict of interest

The authors declare that the research was conducted in the absence of any commercial or financial relationships that could be construed as a potential conflict of interest.

## Publisher’s note

All claims expressed in this article are solely those of the authors and do not necessarily represent those of their affiliated organizations, or those of the publisher, the editors and the reviewers. Any product that may be evaluated in this article, or claim that may be made by its manufacturer, is not guaranteed or endorsed by the publisher.

## Supplementary material

The Supplementary material for this article can be found online at: https://www.frontiersin.org/articles/10.3389/fpsyg.2022.1045947/full#supplementary-material
